# Shotgun Redox Proteomics: Identification and Quantitation of Carbonylated Proteins in the UVB-Resistant Marine Bacterium, *Photobacterium angustum* S14

**DOI:** 10.1371/journal.pone.0068112

**Published:** 2013-07-09

**Authors:** Sabine Matallana-Surget, Ricardo Cavicchioli, Charles Fauconnier, Ruddy Wattiez, Baptiste Leroy, Fabien Joux, Mark J. Raftery, Philippe Lebaron

**Affiliations:** 1 UPMC Univ Paris 06, UMR7621, Laboratoire d’Océanographie Microbienne, Observatoire Océanologique, Banyuls/mer, France; 2 CNRS, UMR7621, Laboratoire d’Océanographie Microbienne, Observatoire Océanologique, Banyuls/mer, France; 3 School of Biotechnology and Biomolecular Sciences, The University of New South Wales, Sydney, Australia; 4 Department of Proteomics and Microbiology, Research Institute for Biosciences Interdisciplinary Mass Spectrometry Center (CISMa), University of Mons, Mons, Belgium; 5 Bioanalytical Mass Spectrometry Facility, The University of New South Wales, Sydney, Australia; Consejo Superior de Investigaciones Cientificas, Spain

## Abstract

UVB oxidizes proteins through the generation of reactive oxygen species. One consequence of UVB irradiation is carbonylation, the irreversible formation of a carbonyl group on proline, lysine, arginine or threonine residues. In this study, redox proteomics was performed to identify carbonylated proteins in the UVB resistant marine bacterium *Photobacterium angustum*. Mass-spectrometry was performed with either biotin-labeled or dinitrophenylhydrazide (DNPH) derivatized proteins. The DNPH redox proteomics method enabled the identification of 62 carbonylated proteins (5% of 1221 identified proteins) in cells exposed to UVB or darkness. Eleven carbonylated proteins were quantified and the UVB/dark abundance ratio was determined at both the protein and peptide levels. As a result we determined which functional classes of proteins were carbonylated, which residues were preferentially modified, and what the implications of the carbonylation were for protein function. As the first large scale, shotgun redox proteomics analysis examining carbonylation to be performed on bacteria, our study provides a new level of understanding about the effects of UVB on cellular proteins, and provides a methodology for advancing studies in other biological systems.

## Introduction

The discovery of the ‘ozone hole’ over Antarctica in the 1980’s [1] prompted research into the environmental impacts of increasing levels of solar ultraviolet radiation, particularly UVB (280–320 nm), reaching the Earth’s surface. The increased UVB irradiation constitutes a potentially important risk to many life forms as this type of radiation damages many different cellular components [2]. Damage includes the dimerization of two adjacent pyrimidine bases located on the same strand of DNA and/or indirect oxidative stress [2]. Oxidative stress arises when reactive oxygen species (ROS) accumulate in cells to the extent that DNA, RNA, lipids and proteins are damaged by oxidation.

**Figure 1:**
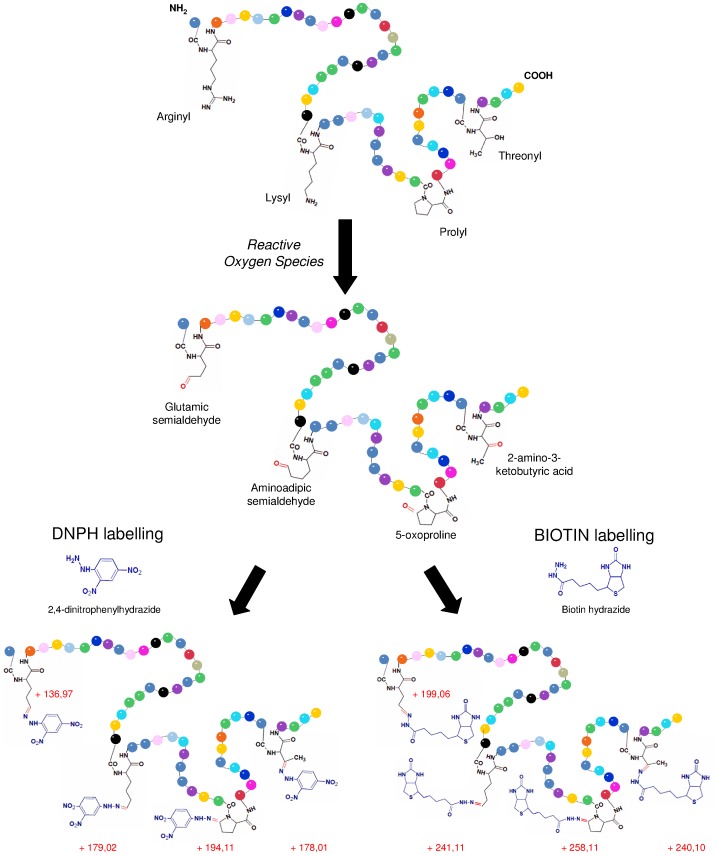
Protein carbonyls produced by direct oxidation of amino acids after reaction with ROS. Carbonyl groups derivatized with DNPH or biotin and the associated increase in molecular weight caused by the modifications are shown in blue.

Carbonylation is the post-translational addition of a carbonyl group (C = O) to the side chains of amino acids and preferentially affects arginine, threonine, proline or lysine (Figure 1), and is caused by diverse oxidative reactions, in particular by direct metal-catalyzed oxidative attack [3]. Carbonyl groups cause changes in protein hydrophobicity, surface charge and associated misfolding of proteins. Oxidative stress often leads to proteins becoming modified by carbonylation [4], [5]. Sulfur-containing amino acids such as cysteine and methionine are also susceptible to ROS. However, these oxidative modifications can be repaired by the cell [3] and most biological systems contain enzymes (disulfide reductases and MeSOX reductases) that can convert the oxidized forms of cysteine and methionine residues, respectively, back to their unmodified forms [3]. In comparison, carbonyl modification is irreversible and therefore causes permanent damage to proteins [6], [7]. For these reasons, protein carbonyl content is widely used as a marker of protein oxidative damage [8], including for age-related disorders [6].

In bacteria, it was recently demonstrated that oxidative damage is the cause, rather than a consequence of radiation-induced cell death. This was demonstrated for both *Escherichia coli* and the radiation resistant bacterium, *Deinococcus radiodurans*, where ionizing radiation resistance was dependent on cellular responses that provided protection against protein carbonylation [9]. Once proteins become carbonylated, they either generate high molecular weight aggregates which are toxic to the cell, or lead to abnormally high rates of protein turnover to remove them from the cell [10]. Estimates based on Western blotting, indicate that approximately 10% of the proteome of *E. coli* is susceptible to carbonylation [11], and we calculated a similar percentage from immunoblots published for *Saccharomyces cerevisiae* [12], and other immunoblots for *E. coli* [13]. Sequence composition denoting potential hotspots for carbonylation have also been reported [11], although the predictive value of these hotspot sequences has been questioned [14].

*Photobacterium angustum* S14 is a marine heterotrophic gram-negative bacterium isolated from surface coastal waters in Botany Bay (Sydney), Australia and its genome has been fully sequenced [15]. *P. angustum* has served as a model organism for diverse stress studies, such as starvation and UV radiation [16], [17], [18], [19], [20]. Importantly, *P. angustum* was found to be resistant to long-term UVB radiation and this capacity was linked to a very efficient mechanism of DNA damage repair [19], [20]. The development of proteomic methods for studying the adaptive responses of *P. angustum* have proven valuable [16], [21], including the use of two-dimensional difference gel electrophoresis (2D DIGE) and Isotope Coded Protein Labeling (ICPL) for examining UVB biomarkers [20].

“Redox proteomics” refers to the proteomic analysis of the oxidative stress-induced modification of proteins [22], [23], [24]. A recent redox proteomics study used a shotgun mass spectrometry based approach to characterize thiol-modifications, following oxidation with hypochloric acid in *Bacillus subtilis* [24]. A technique using biotin hydrazide has been described that combines an enrichment of the carbonylated proteins [25], [26], [27], [28], [29] with iTRAQ quantitative proteomic labeling [25], [29]. A recent study used the DNPH coupled with mass spectrometry (MALDI and ESI-MS) to identify the carbonylation sites of β-lactoglobulin and bovine serum albumin (BSA) [30]. While carbonylated proteins can be quantified by immunoblotting [31], the accurate mapping of each protein differentially oxidized between different conditions is not possible.

In the present study, we performed redox proteomics to define the global protein targets for carbonylation as a means of assessing the cellular response, at the protein level, to UVB induced oxidative damage. We used one of the latest generations of highly sensitive mass spectrometers, an AB SCIEX TripleTOF™ 5600, employing label-free methods applied to several biological replicates of two test conditions, thereby ensuring we obtained a comprehensive assessment of carbonylation. A UVB dose was used that was ecologically relevant (6.6 kJ m^−2^), and had previously been adopted for proteomic and physiological studies of *P. angustum* [19], [20], enabling comparative assessments of the data to be made. Carbonyls were identified, quantified and their locations in proteins determined using two derivatization methods linked to mass spectrometry analysis. To tag the carbonylated proteins, biotin hydrazide and dinitrophenylhydrazide (DNPH) were used. In our study, we developed a new approach that combined shotgun proteomics with DNPH derivatization and label-free quantitation that proved very successful for studying protein carbonylation. The use of DNPH to react with carbonyl functional groups was first described in the 1930s [32], but had not been used for quantitative shotgun proteomics studies.

## Materials and Methods

### Bacterial Growth Conditions and UVB Radiation Treatment

*P. angustum* S14 was grown aerobically at 25°C in the dark (rotary shaker, 130 rpm) in 500 mL of Artificial Sea Water (ASW) medium supplemented with 3 mM D-glucose (ASW-G), vitamins and trace elements [33]. Growth was monitored by optical density at 620 nm (OD_620_). At the beginning of the mid-log phase (OD = 0.1), cells were harvested and divided into two equal 120 mL volumes and placed into 250-mL quartz flasks with flat tops for subsequent treatment by UVB irradiation or maintenance in the dark as a control. For UVB treatment, flasks were covered with cellulose acetate (50% transmission at 280 nm) to remove residual UVC and exposed to UVB lamps (30 W/312 nm, Vilbert Lourmat, Fisher Scientific) to generate a total UVB dose of 1.05 W m^−2^. Irradiated cells and dark controls were mixed with continuous magnetic stirring (100 rpm) for 1.75 h (corresponding to a UVB dose of 6.6 kJ m^−2^) at 25°C. At the end of this period, the OD_620_ was measured, and the cells were harvested for proteomics. The entire volume of the cultures from both conditions was centrifuged at 8 000 *g* for 15 min at 4°C, and then stored at -80°C until use. For carbonyl enrichment using biotin labeling two biological replicates of each growth condition (UVB and dark) were used, and for DNPH derivatization, three biological replicates were used.

### Protein Extraction and Quantification

Pelleted cells were resuspended in 300 µL of 10 mM Hepes Buffer (pH 8) containing 6 M Urea and 2 M Thiourea. Cells were broken by sonication on ice using a digital Branson sonifier (amplitude 45%, 5 cycles of 10 s, 1 pulse rate). The samples were subsequently centrifuged at 8 000 *g* at 4°C for 10 min and the supernatant containing soluble proteins was preserved at −80°C. The protein concentration was determined using the Bradford method according to the manufacturer’s instructions, using bovine *γ*-globulin as a protein standard (Bio-Rad Protein Assay kit, Bio-Rad, Hertfordshire, UK).

### Enrichment of Carbonylated Proteins by Biotin/Avidin Chromatography

Protein samples were incubated at room temperature for 2 h with 5 mM of biotin hydrazide with gentle shaking and samples cooled on ice for 5 min. The amine of hydrazide (–NH-NH_2_) reacts specifically with the carbonyl functional groups in aldehydes and ketones to form hydrazone bonds. Subsequently, an equal volume of 30 mM of sodium cyanoborohydride in Phosphate Buffered Saline (PBS; 0.1 M sodium phosphate, 0.15 M NaCl) was added for 1 h on ice, stabilizing the hydrazone bonds formed between the biotin and carbonyl groups. The sample was washed three times through an AMICON Ultra-15 3 kD Centrifugal filter device against 5 mL of PBS to remove excess biotin hydrazide [34]. Biotinylated proteins were purified using a monomeric avidin column as described in the Pierce Monomeric Avidin Kit. Briefly, samples were incubated 1 h in the monomeric avidin column, washed with PBS, eluted with biotin (2 mM) and regenerated with 0.1 M glycine. After the avidin chromatography, the eluted fraction was concentrated (AMICON Ultra-15 3 kD) to a final volume of 500 µL. Purified proteins were separated by SDS-PAGE and visualized by silver staining (GE Healthcare). Subsequently, for identification purposes, proteins samples were reduced with 25 mM of dithiotreitol for 30 min at 60°C and alkylated with iodoacetamide (100 mM) for 30 min at 25°C. Proteins were digested with a solution of trypsin with a ratio of 1/50 (enzyme/substrate) overnight at 37°C.

### DNPH Derivatization for Label-free Quantitative Proteomics

Proteins were reduced with 500 mM of DTT and derivatized with 2 mg mL^−1^ DNPH in 2 N HCl for 1 h in the dark. Similar to biotin hydrazide derivatization, DNPH reacts with the carbonyl functional groups in aldehydes and ketones, forming the corresponding 2,4-DNP-hydrazone [32]. Proteins samples were precipitated with 20% trichloroacetic acid for 3 h at 4°C and centrifuged for 10 min at 8 000 *g* at 4°C. Protein pellets were washed two times with 1 mL ethanol/ethyl acetate (v/v) to remove excess DNPH. Subsequently, samples were resuspended in 110 µL of protein resolubilization buffer (6 M guanidine hydrochloride; 0.5 M KH_2_PO_4_, pH 2.5) at 37°C for 15 min with gentle shaking. To estimate protein loss and determine the amount of enzyme needed for protein digestion, a Bradford assay was performed on each sample. Subsequently, protein samples were reduced, alkylated and digested, as described above.

### Liquid Chromatography Tandem Mass Spectrometry (LC-MS/MS)

Protein identification and quantification was performed using a label-free strategy on a UHPLC-HRMS platform composed of an eksigent 2D liquid chromatograph and an AB SCIEX TripleTOF™ 5600. Peptides were separated on a 25 cm C18 column (Acclaim pepmap100, 3 µm, Dionex) by a linear acetonitrile (ACN) gradient [5–35% (v/v), in 15 or 120 min for short and long runs, respectively] in water containing 0.1% (v/v) formic acid at a flow rate of 300 nl min^−1^. In order to reach high retention stability, which is a requirement for label-free quantification, the column was equilibrated with a 10x volume of 5% ACN before each injection. Eluant was sprayed using the Nanospray Source into the TripleTOF™ 5600. Mass spectra (MS) were acquired across 400–1500 m/z in high resolution mode (resolution >35000) with 500 ms accumulation time. The instrument was operated in DDA (data dependent acquisition) mode and MS/MS were acquired across 100–1800 m/z. For short runs, precursor selection parameters were: intensity threshold 400 cps, 20 precursors maximum per cycle, 100 ms accumulation time, 10 s exclusion after one spectrum. For long runs, precursor selection parameters were: intensity threshold 200 cps, 50 precursors maximum per cycle, 50 ms accumulation time, 30 s exclusion after one spectrum. A long run procedure was used to acquire quantitative data, and a duty cycle of 3 sec per cycle was used to ensure that high quality extracted ion chromatograms (XIC) could be obtained.

Protein searches were performed against a local copy of the *P. angustum* database (retrieved from NCBI on December 19^th^, 2007; 4558 proteins) using either Mascot (for biotin analysis) or ProteinPilot Software v4.1 (for DNPH). Search parameters included differential amino acid mass shifts for oxidized methionine (+16 Da), and either DNPH or biotin hydrazide-labeled carbonylated residues, with mass shifts appropriate for commonly known carbonyl groups. Two missed internal tryptic cleavage sites were accounted for in the search parameters, enabling the identification of peptides carrying biotinylated carbonyl modifications that trypsin did not recognize. Differential mass shifts of 241.11, 258.12, 199.06, and 240.10 Da were used for lysine modified to aminoadipic semialdehyde, proline to 5-oxo-proline, arginine to glutamic semialdehyde and threonine to 2-amino 3-ketobutyric acid modifications labeled with biotin hydrazide, respectively. For DNPH derivatization, the mass shifts used for the adducts of lysine, arginine, threonine and proline, were 179.02; 136.97, 178.01 and 194.11 Da, respectively (Figure 1). Mass tolerance was set to 15 ppm in MS and 0.05 Da in MS/MS.

For quantification, the quant application of PeakView was used to calculate XIC for all peptides identified with a confidence >0.99 using ProteinPilot™. A retention time window of 2 min and a mass tolerance of 0.015 m/z were used. The area under the curve was exported in MarkerView™, in which they were normalized based on the summed area of the entire run. MarkerView™ enabled an average intensity for UVB and dark conditions to be calculated, as well as the significance of the difference between conditions based on a student t-test. Quantified proteins were kept with a p value <0.1 and with at least one peptide quantified with a p value <0.1.

### Computational Analyses

All proteins identified by proteomics were classified into Cluster of Orthologous Groups (COG) (http://bioinfo.biotec.or.th). Structural data of the proteins were downloaded from the Protein Data Bank. Visualization of 3D protein structure, amino acids positions, and distances between carbonylated residues were estimated using Cn3D software v4.3.

## Results

Redox proteomics was performed by either enriching the relatively low abundance carbonylated proteins using biotin tagging and avidin affinity chromatography, or by DNPH derivatization of carbonyl functional groups without enrichment. Carbonylated proteins were then identified by performing LC-MS/MS analysis to identify peptides with either biotin hydrazide- or DNPH-derivatized carbonylated residues.

### Biotin Labeling

**Figure 2:**
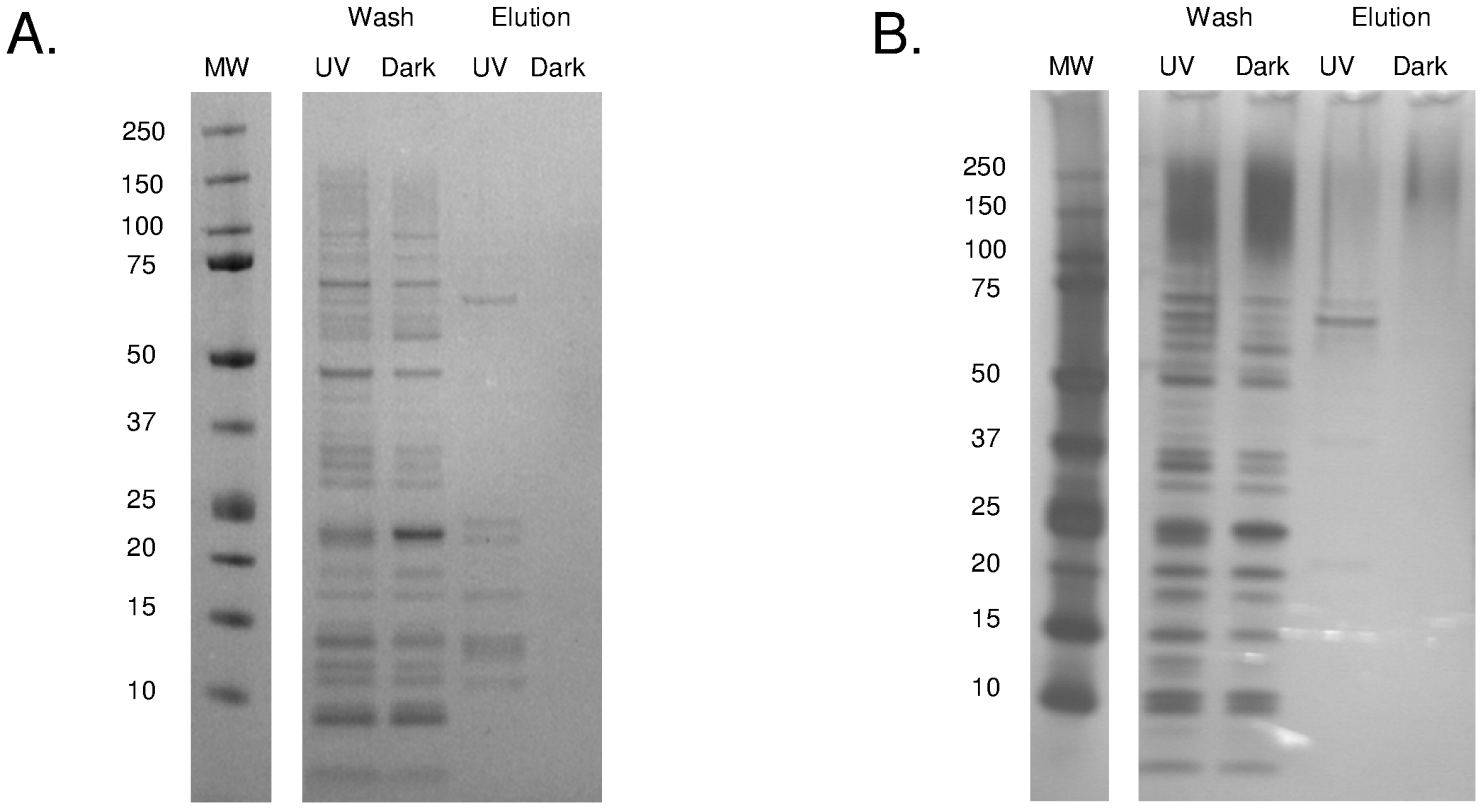
SDS-PAGE of proteins purified by avidin affinity chromatography. Replicates (A and B) of proteins extracted from cells exposed to UV or darkness, electrophoresed on 4–12% acrylamide gradient gels and visualized by silver staining. MW: molecular weight marker (kDa).

Following derivatization with biotin hydrazide and capture on an immobilized avidin column, biotinylated proteins were eluted with biotin and visualized using SDS-PAGE. SDS-PAGE analysis of two replicate treatments revealed many more silver stained protein bands for the eluted fractions of UVB compared to dark conditions where no bands were detected (Figure 2). LC-MS/MS analysis of the eluted fractions led to the identification of a total of 199 proteins; 141 and 58 for the UVB and dark treatments, respectively, with 45 proteins in common between the two conditions. However, while protein identity was able to be determined for 199 proteins eluted from the avidin column, only three peptides corresponding to three proteins were detected with biotinylated residues (UVB treatment: VAS14_16581_serine hydroxymethyltransferase and VAS14_17649_hypothetical O methyltransferase; Dark treatment: VAS14_17649_hypothetical O methyltransferase; Tables S1 and S2). The reasons for the lack of identification of biotinylated residues are considered in the Discussion, *Limitations of biotin-avidin enrichment.*

All identified proteins were classified into COG categories (Table 1). A larger number of COG categories were represented by proteins from UVB treatment (19 COG categories) compared to the dark control (14 COGs). In both UVB and dark treatments, proteins in the Translation, Ribosomal Structure and Biogenesis category (COG J) were the most abundant, with 49 and 22 carbonylated proteins for UVB and dark, respectively. COG categories unique to UVB were Intracellular trafficking, secretion, and vesicular transport (COG U), Energy Production and Conversion (COG C), Nucleotide transport and metabolism (COG F), Coenzyme transport and metabolism (COG H), Cell cycle control (COG D).

**Table 1:** COG distribution of carbonylated proteins identified following biotin labeling.

COG categories		UVB		DARK	
		Nb of prot.	% of prot	Nb of prot.	% of prot
[COG J]	Translation, ribosomal structure and biogenesis	47	33	22	39
[COG K]	Transcription	5	3	2	4
[COG L]	Replication, recombination and repair	5	3	2	4
[COG D]	Cell cycle control, cell division, chromosome partitioning	1	1	0	0
[COG T]	Signal transduction mechanisms	4	3	3	5
[COG M]	Cell wall/membrane/envelope biogenesis	7	5	3	5
[COG N]	Cell motility	2	1	1	2
[COG U]	Intracellular trafficking, secretion, and vesicular transport	4	3	0	0
[COG O]	Posttranslational modification, protein turnover, chaperones	14	10	5	9
[COG C]	Energy production and conversion	7	5	0	0
[COG G]	Carbohydrate transport and metabolism	7	5	3	5
[COG E]	Amino acid transport and metabolism	7	5	6	11
[COG F]	Nucleotide transport and metabolism	1	1	0	0
[COG H]	Coenzyme transport and metabolism	3	2	0	0
[COG I]	Lipid transport and metabolism	6	4	1	2
[COG P]	Inorganic ion transport and metabolism	5	3	1	2
[COG Q]	Secondary metabolites biosynthesis, transport and catabolism	3	2	1	2
[COG R]	General function prediction only	6	4	3	5
[COG S]	Function unknown	9	6	4	7

Numbers indicate the number of proteins in each COG category.

### Carbonylated Proteins Identified by DNPH Derivatization

A total of 1221 proteins were identified (UVB/dark) by LC-MS/MS, including 405 new proteins not previously described [20] bringing the total number of proteins identified for *P. angustum* S14 using MS/MS methods to 1398 (∼31% of the theoretical proteome). A total of 138 proteins were quantified (p value <0.05) demonstrating fold changes of 0.02 to 4.83. Sixty two non-redundant carbonylated proteins (45 and 36 from UVB and dark treatments, respectively) and 117 non-redundant peptides were identified, of which 19 proteins were in common between the two conditions (Table S3). More predicted membrane proteins were carbonylated following dark treatment (6 proteins) compared to UVB (2 proteins) or either treatment (2 proteins) (Table S3). Because a triple TOF mass spectrometer was used with three biological replicates of UVB and dark grown cells, it is likely that we detected a significant number of carbonylated proteins in *P. angustum* that were present under the growth conditions tested.

COG category C (Energy Production and Conversion) and J (Translation, Ribosomal Structure and Biogenesis) represented the largest number of DNPH derivatized proteins for the UVB (Table 2) and dark treatments (Table 3), respectively. COG U (Intercellular trafficking secretion) was only represented for UVB samples. From the 62 non-redundant proteins, only 4 were annotated as proteins with unknown functions.

**Table 2:** Carbonylated proteins identified by DNPH derivatization following UVB treatment.

Proteins name	COG	Nb of pept (>95% conf)	Sequence of carbonylated peptides
VAS14_01896 putative DnaK-related protein	O	1	DLDNRLT**K**AAK
VAS14_09599 acetoacetyl-CoA reductase	IQR	4	MSKVALV**T**GAKGGIGSSITQALVDAGFR
			VVATYYPTGE**K**AAQEWLAANNYSSESVR
			LALPQEVAAAVTFLASDAAAYI**T**GETLSVNGGLYMQ
			IGSSI**T**QALVDAGFR
VAS14_09604 acetyl-CoA acetyltransferase	I	76	GALAE**T**SVPVEAIDEVIFGNVVGAGQGMGPGR
			GALAETSV**P**VEAIDEVIFGNVVGAGQGMGPGR
			MGNLELSDLLIADGL**T**DAFNNYHMGVTAENVVEK
			GALAE**T**SVPVEAIDEVIFGNVVGAGQGMGPGR
			YNL**T**PLAEIESYAQAGIAPEIMGLGPVPAVLK
VAS14_09609 hypothetical protein	/	1	APYVKFN**K**LFTKNVEELTELQLAAVR
VAS14_10584 3-deoxy-7-phosphoheptulonate synthase	E	1	LLLDLTESGL**P**TAGEFLDMITPQYMGDLISWGAIGAR
**VAS14_14994 transketolase**	G	5	TPGHPEYGYAPGVETT**T**GPLGQGITNAVGMALAEK
			TPGHPEYGYAPGVET**T**TGPLGQGITNAVGMALAEK
			**T**PGHPEYGYAPGVETTTGPLGQGITNAVGMALAEK
VAS14_15214 ATP synthase subunit B	C	2	MPSAVGYQ**P**TLAEEMGVLQER
**VAS14_04158 arginine ABC transporter**	ET	2	FAMEATYA**P**FEYMDENNQIQGFDVDIAK
			VGVQNGSTHQSYLTDQMPGV**T**AVPYTSYQDAFIDMK
VAS14_04998 elongation factor EF-2	J	2	AGPQLLE**P**IMHVDVFTPEDHVGDVIGDLNR
			GMQLVLDAVVDYLPSPTEVDPQPLTDPETGEPTGEVA**T**VSADEPLK
VAS14_05433 isocitrate dehydrogenase	C	1	FTEGAF**K**DWGYEVALQEFGAELLDGGPWMTLK
VAS14_05968 hypothetical protein	/	3	SDVSNNTLTTE**P**ETCTVEFTQTSGAIDMSNSK
VAS14_06363 glycosyl transferase	/	1	KESR**KK**LFNDESSSPWVLFNTLSK
VAS14_06513 formate acetyltransferase	C	19	TPEYDELFSGD**P**IWATESMGGMGLDGR
			TPEYDELFSGDPIWA**T**ESMGGMGLDGR
VAS14_00841 putative pyruvate kinase II	G	1	GGGLSAEALTDKD**K**ADILTAAAMGVDYLAVSFPR
VAS14_16526 bifunctional GMP synthase/glutamine amidotransferase	F	1	VAETETC**P**FAAMANEEK
**VAS14_16581 serine hydroxymethyltransferase**	E	1	TLAG**P**RGGLILSNEGEDLYKK
VAS14_19626 putative MreB, Actin-like ATPase	D	1	SIDLGTANTLIYV**K**GQGIVLDEPSVVAIR
VAS14_19926 translocase	U	1	VQ**R**E**R**FFAVVDEVDSILIDEAR
**VAS14_19986 putative Pyruvate dehydrogenase complex**	C	4	LMPEFWQF**P**TVSMGLGPISAIYQAR
VAS14_20006 aconitate hydratase	C	4	ALVELLKN**P**PAGEESVLLDLLENR
			FPLGISFPAGSGLVAFAAATGVM**P**LDMPESILVR
			FPLGISFPAGSGLVAFAAA**T**GVMPLDMPESILVR
			IAPIFFN**T**MEDAGALPIEVDVTQLAMGDVIDVYPFK
VAS14_20221 S-adenosylmethionine synthetase	H	1	EQGAGDQGIMFGYA**T**NETEVFMPAPITYSHR
VAS14_20236 phosphoglycerate kinase	G	38	SASDIAADDMVLDLG**P**DSAQALADIIMNAK
VAS14_20256 Phosphoglycerate dehydrogenase	H	1	GIPVFNAPFSN**T**RSVAELVLGELLLLLR
**VAS14_07384 ompL_phopr porin-like protein L precursor**	M	1	ADGSLGML**T**DVTDIMAYAGSVVGSTK
VAS14_07404 carbamoyl-phosphate synthase large subunit	E	1	EDGYETIMVNCN**P**ETVSTDYDTSDR
VAS14_07699 bifunctional aspartokinase I/homeserine dehydrogenase I	E	2	QAARLSR**R**KFMYDTTVGAGLPVIENLQNLLAAGDELQR
VAS14_07734 putative glutamate synthase, large subunit	E	1	IQGLTIDDIAQEVLV**R**
**VAS14_21322 tryptophanyl-tRNA synthetase**	J	1	PIVLSGVQ**P**SGELSIGNYLGALR
VAS14_21427 phosphoenolpyruvate carboxylase	C	4	IEQ**R**LEQLIAQAWHSDVIR
			QQEELP**P**ALEEALMVTIAGIATGMR
**VAS14_21532 6-phosphofructokinase**	G	1	GGQ**PT**AFDRVLASRMGAYAVDLLQQGEGGR
VAS14_22994 glutamine synthetase	E	10	AEIP**T**VAESLQGALQALSDDR
			IHPGEAMDKDLYDLPAEEAAEI**PT**VAESLQGALQALSDDR
			IHPGEAMD**K**DLYDLPAEEAAEI**P**TVAESLQGALQALSDDR
			IHPGEAMDKDLYDL**P**AEEAAEI**P**TVAESLQGALQALSDDR
			IHPGEAMD**K**DLYDLPAEEAAEIP**T**VAESLQGALQALSDDR
			FGDPAAN**P**YLAFAAMLMAGLDGIQNK
VAS14_23029 phosphoenolpyruvate carboxykinase	C	1	AEE**T**KTELKGFEKGIVTELGAVAVDTGIFTGR
VAS14_22357 DNA gyrase subunit B	L	3	LSTEMMAEEAQVEAWLT**P**LIAALNAK
VAS14_18544 30S ribosomal protein S1	C	2	**R**HEAWIQLE**K**AYEDAETVVGIINGK
			**T**ESFAQLFEESLNQVETRPGAIVK
VAS14_18941 chaperonin GroEL	J	2	AGDANYGYNAA**T**GEYGDMIEMGILDPTK
			ENT**T**IIDGSGEEAMIQGR
**VAS14_19156 DNA-directed RNA polymerase beta subunit**	O	14	EQEVYMGEIPLM**T**DNGTFVINGTER
			EQEVYMGEI**P**LMTDNGTFVINGTER
VAS14_19166 50S ribosomal protein L10	K	3	AVEG**T**DFECLQDVFVGPSLIGFSNEHPGAAAR
VAS14_18809 adenylosuccinate synthetase	I	1	SGEILEVS**P**MAADEYEDLELVYETMPGWSETTFGAK
VAS14_22247 ketol-acid reductoisomerase	F	5	GETAETQFENY**P**SSDIQISEQEYFDNGILMVAMVR
VAS14_07339 transcription elongation factor NusA	H	1	**P**RERIFEALEIALATATK
VAS14_07344 hypothetical protein	K	1	MTALETQLTEMLE**P**SVLALGYELVGLEFIR
VAS14_19296 putative ribosomal subunit protein S5	S	4	HTGSQVYMQ**P**ASEGTGIIAGGAMR
VAS14_19336 DNA-directed RNA polymerase alpha subunit	K	2	MQGSVTEFLKP**R**LVDIEQVSTTHAK
			ETNGTLDPEEAIRRAA**T**ILAEQLDAFVDLR
VAS14_16399 peptide chain release factor 1	J	1	LNEVMEGDLDALIQPVF**T**EYQADQLAAMSEQN
**VAS14_19191 elongation factor Tu**	J	25	ELLSEYDF**P**GDDCPVIMGSALGALNGEK
			ELLSEYDFPGDDC**P**VIMGSALGALNGEK
			NMI**T**GAAQMDGGILVVAATDGPMPQTR
			NMITGAAQMDGGILVVAATDG**P**MPQTR

(*) Bold proteins indicate that carbonylation is in a key functional domain. Bold residues indicate sites of carbonylation.

**Table 3:** Carbonylated proteins identified by DNPH derivatization following dark treatment.

Proteins name	COG	Nb of pept (>95% conf)	Sequence of carbonylated peptides
VAS14_09599 acetoacetyl-CoA reductase	IQR	2	VALVTGA**K**GGIGSSITQALVDAGFR
VAS14_09604 acetyl-CoA acetyltransferase	I	34	GALAE**T**SVPVEAIDEVIFGNVVGAGQGMGPGR
			YNL**T**PLAEIESYAQAGIAPEIMGLGPVPAVLK
			PLAEIESYAQAGIAPEIMGLGPVPAVLKALD**K**
VAS14_10219 phosphoenolpyruvate synthase	G	1	Y**T**LWFNTLSMNDVNQVGGK
VAS14_10584 3-deoxy-7-phosphoheptulonate synthase	E	3	LLLDLTESGL**P**TAGEFLDMITPQYMGDLISWGAIGAR
**VAS14_14994 transketolase**	G	3	TPGHPEYGYAPGVETT**T**GPLGQGITNAVGMALAEK
**VAS14_04153 putative arginine ABC transporter, ATP-bindingprotein**	E	1	ALMMKPEVLLFDE**P**TAALDPEITSQIVQIIK
**VAS14_04158 arginine ABC transporter**	ET	3	FAMEA**T**YAPFEYMDENNQIQGFDVDIAK
			FAMEATYA**P**FEYMDENNQIQGFDVDIAK
VAS14_04998 elongation factor EF-2	J	2	TGEVHDGES**T**TDFMEQEAER
VAS14_05363 seryl-tRNA synthetase	J	1	PAQETY**R**EISSCSNMWDFQAR
**VAS14_05498 putative oligopeptide ABC transporter**	E	1	TASPYASYIQMT**T**MANAEDIIAGK
VAS14_05968 hypothetical protein	/	15	SDVSNNTLTTE**P**ETCTVEFTQTSGAIDMSNSK
**VAS14_06493 putative amino acid ABC transporter**	T	1	GVKIGVQRAT**T**HDKYLTDNFGESVEIVR
VAS14_06513 formate acetyltransferase	C	19	TPEYDELFSGD**P**IWATESMGGMGLDGR
			TPEYDELFSGDPIWA**T**ESMGGMGLDGR
VAS14_01951 hypothetical protein	S	1	A**T**MSLVREQELGQPFQFDNFRD
VAS14_17071 flagellin	N	1	H**T**MSNLANINENVNASNSR
**VAS14_18001 putative alcohol dehydrogenase/acetaldehyde dehydrogenase**		1	SDLEGKKRAFLVTD**R**FLFNNGYADEIVSLLK
**VAS14_19986 putative Pyruvate dehydrogenase complex**	C	1	LMPEFWQF**P**TVSMGLGPISAIYQAR
VAS14_20006 aconitate hydratase	C	3	IA**P**IFFNTMEDAGALPIEVDVTQLAMGDVIDVYPFK
			FPLGISFPAGSGLVAFAAATGVMPLDMPESILV**R**
VAS14_20236 phosphoglycerate kinase	G	37	SASDIAADDMVLDLG**P**DSAQALADIIMNAK
**VAS14_07384 ompL_phopr porin-like protein L precursor**	M	2	VDATYYFNSNF**R**TYASYTFNMLDK
VAS14_21207 putative FKBP-type peptidyl-prolyl cis-trans isomerase 1	O	1	TPAADAKVEF**K**TEDQ**K**AAYAIGASLAQYLSANLDQQK
VAS14_21577 phosphoglyceromutase	G	1	LSDLAPTMLSL**T**DMEIPAEMSGQVLYNLK
VAS14_22994 glutamine synthetase	E	2	FGDPAAN**P**YLAFAAMLMAGLDGIQNK
			GINESDMVMM**P**DASSAVLDPFTEDATLNIR
VAS14_22492 ATP synthase subunit D	C	1	AQWAEMLNFASEVAKND**T**MQDVLDSGFAAEK
VAS14_22497 ATP synthase subunit A	C	2	IHGLADVMQGEMIEL**P**GGLFALALNLER
			TALAQY**R**ELAAFAQFSSDLDDATK
**VAS14_22552 branched-chain amino acid aminotransferase**	H	1	MDLIVAAF**P**WGSYLGEEALENGVDAMISSWNR
VAS14_18941 chaperonin GroEL	O	2	AGDANYGYNAA**T**GEYGDMIEMGILDPTK
VAS14_19156 DNA-directed RNA polymerase beta subunit	K	8	EQEVYMGEI**P**LMTDNGTFVINGTER
VAS14_18764 50S ribosomal protein L9	J	1	AGDEGKLFGSIG**T**RDIADAVTAAGVALVK
VAS14_18809 adenylosuccinate synthetase	F	1	SGEILEVS**P**MAADEYEDLELVYETMPGWSETTFGAK
VAS14_22247 ketol-acid reductoisomerase	H	6	GETAETQFENY**P**SSDIQISEQEYFDNGILMVAMVR
VAS14_07124 molecular chaperone DnaK	O	1	KDVN**P**DEAVAVGAAVQGGVLAGDVK
VAS14_19286 50S ribosomal protein L6	J	1	AECPSQ**T**EIVLTGTDK
VAS14_19296 putative ribosomal subunit protein S5	J	8	HTGSQVYMQ**P**ASEGTGIIAGGAMR
VAS14_19331 30S ribosomal protein S4	J	2	TAA**R**IKGNTGENLLQLLEGR
**VAS14_19191 elongation factor Tu**	J	25	ELLSEYDF**P**GDDCPVIMGSALGALNGEK
			GTVVTGRVEQGII**T**VGDEVEIVGIVDTIK

(*) Bold proteins indicate that carbonylation is in a key functional domain. Bold residues indicate sites of carbonylation.

Among the 19 DNPH derivatized proteins in common to both treatments, 11 were robustly (p value <0.1 and at least one peptide quantified with a p value <0.1) quantified (Table 4). While a p value <0.05 can be adopted as a stringent value for quantitative proteomics (*e.g.* 20), we found that reproducibility of quantifications of carbonylated peptides across all three biological replicates was low. We reasoned that this may indicate there is a degree of chance associated with which residues become carbonylated, and therefore judged the criteria to be inappropriate. Four proteins did have peptides that were quantitated with p values <0.05 (Table 4), indicating that carbonylation occurs more reproducibly at those sites.

**Table 4:** Quantification of carbonylated proteins and peptides and location of the modification.

Proteins name	Protein ratio(UV/DARK)	p val prot.	Nb of total identifiedpept. (>95%)	Nb of totalquantified pept.(pval <0.1)	Carbonylated Peptide ratio(UV/DARK)	p val pept.	Sequence of carbonylated peptides
VAS14_09599 acetoacetyl-CoA	0.8	0.07	81	10	0.39	0.09	MSKVALV**T**GAKGGIGSSITQALVDAGFR
eductase*					0.69	0.44	VVATYYQTGE**K**AAQEWLAANNYSSESVR
VAS14_14994 transketolase*	0.96	0.10	60	5	0.26	0.19	**T**PGHPEYGFAPGVETTTGPLGQGITNAVGMALAEK
					0.77	0.08	TPGHPEYGYAPGVET**T**AGPLGQGITNAVGMALAEK
VAS14_04158 arginine ABC	0.51	0.05	81	11	0.75	0.78	FAMEATYA**P**FEYVDENNQIQGFDVDIAK
transporter					2.56	0.06	FAMEA**T**YAPFERMDENNQIQGFDVDIAK
VAS14_04998 elongation factor EF-2	1.26	0.09	151	13	0.76	0.65	TGEVHDGEA**T**TDFMEQEAER
					2.12	0.36	GMQLVLDAVVDYLPSPTEVDPQPLTDPN**T**GEPTGEVATVSADEPLK
					5.21	0.07	AGPQLLE**P**IMNVDVFTPEDHVGDVIGDLNR
VAS14_05968 hypothetical protein*	0.74	0.1	46	3	0.79	0.09	SDVSNNTLTTE**P**ETATVEFTQTSGAIDMSNSK
VAS14_06513 formate	0.49	0.08	132	23	0.15	0.01	TPEYDELFSGD**P**IWATESMGGMGLDGR
acetyltransferase*					0.16	0.01	TPEYDELFSGDPIWA**T**DSMGGMGLDGR
					0.31	0.01	TPEYDELFSGD**P**IWATESMGGMGLDGR
VAS14_20236 phosphoglycerate	0.57	0.04	75	3	0.47	0.01	SASDIAADDMVLDLG**P**DSAQALADIIMNAK
kinase*							
VAS14_07384 ompL_phopr porin-like	1.11	0.08	73	15	4.53	0.02	ADGALGML**T**DVTDIMAYAGSVVGSTK
protein L precursor*							
VAS14_22994 glutamine synthetase	0.6	0.08	130	19	0.04	0.01	GINERDMVMM**P**DASSAVLDPFTEDATLNIR
					0.41	0.68	IHPGEAMDKDLYDL**P**AEEAAKIPTVAESLQGALQALSDDR
					0.5	0.004	FGDPAAN**P**YLAFAAMLMAGLDGIQNK
					0.58	0.58	IHPGEAMD**K**DLYDLPAEEAAEIPTVVESLQGALQALSDDR
					2.47	0.3	IHPGEAMDKDLYDLPAEEAAEI**PT**LAESLQGALQALSDDR
					2.65	0.25	IHPGEAMQ**K**DLYDLPAEEAAEI**P**TVAESLQGALQALSDDR
					2.69	0.3	AEIP**T**VAESLQGALQALSDDR
VAS14_19296 putative ribosomal	1.45	0.02	41	5	0.14	0.01	HTGSQVYVQ**P**ASEGTGIIAGGAM[DTM]R
subunit protein S5							
VAS14_19191 elongation factor Tu	1.32	0.05	200	25	0.04	0.03	ELLSEYDFPGDDA**P**VIMGSALGALNGEK
					6.12	0.01	ELLSEYDFPGDDA**P**VIMGSALGALNGEK

Protein ratio refers to the differential abundance of the protein between UVB and dark treatments. Peptide ratio refers to the differential abundance of the carbonylated peptides. Peptides were scored as either carbonylated or not irrespective of whether multiple residues in the peptide were carbonylated. Data is for the combination of all replicates.

* Stars show proteins with similar trends for peptide and protein ratios (UVB/dark ratio <1 or >1). Bold residues in the sequence of peptides indicate sites of carbonylation.

The differential peptide abundance (UVB/dark) of carbonylated peptides ranged from 0.04 to 6.12, and the differential protein abundance (UVB/dark) ranged from 0.49 to 1.45. Six of the 11 proteins had similar trends for both protein and carbonylated peptide content (Table 4). Five had a UVB/dark ratio <1 (VAS14_09599 acetoacetyl-CoA reductase, VAS14_14994 transketolase, VAS14_05968 hypothetical protein, VAS14_06513 formate acetyltransferase, VAS14_20236 phosphoglycerate kinase) and one had a UVB/dark >1 (VAS14_07384 ompL_phopr porin-like protein L precursor). Three of the five proteins that had a UVB/dark ratio <1 were transferases. The ratio of UVB/dark protein and carbonylated peptide abundances varied for the other five proteins (VAS14_22994 glutamine synthetase, VAS14_19296 putative ribosomal subunit protein S5, VAS14_19191 elongation factor Tu, VAS14_04158 arginine ABC transporter, VAS14_04998 elongation factor EF-2) (Table 4). The total number of carbonylated plus non-carbonylated peptides was high (41–200) for each of the carbonylated proteins that were quantified (Table 4).

### Characteristics of Carbonylation

**Figure 3:**
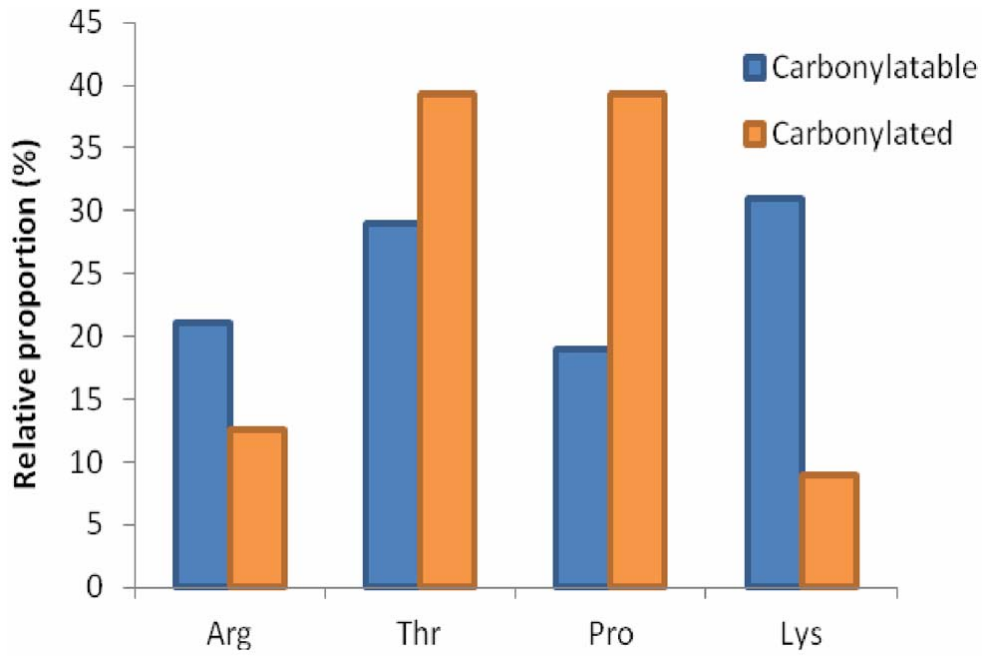
Relative proportion of R, T, P and K residues that were carbonylated. Proportion of each residue as a fraction of the total R+T+P+K content in the 62 carbonylated proteins *P. angustum* (blue), and relative proportion of those residues that were carbonylated (orange). Comparing the percentage that each residue represents versus the percentage that was experimentally determined to be carbonylated highlights the disproportionately high level of carbonylation of P residues.

In the 62 proteins detected using DNPH derivatization, residues that were potentially capable of being irreversibly carbonylated (*i.e.* R, K, P and T) represented 19.7% of the amino acid composition, and were present in the proteins with a relative abundance of K>T>R >P. A similar proportion of R+K+P+T (*i.e.* ∼20%) was present in the total proteome (4558 proteins) and the 1221 identified proteins. The extent of carbonylation detected reflected a different order, with P and T (39%) more frequently modified than R (12%) or K (9%) (Figure 3), indicating that despite having the lowest relative abundance in proteins, proline was relatively frequently carbonylated.

**Figure 4:**
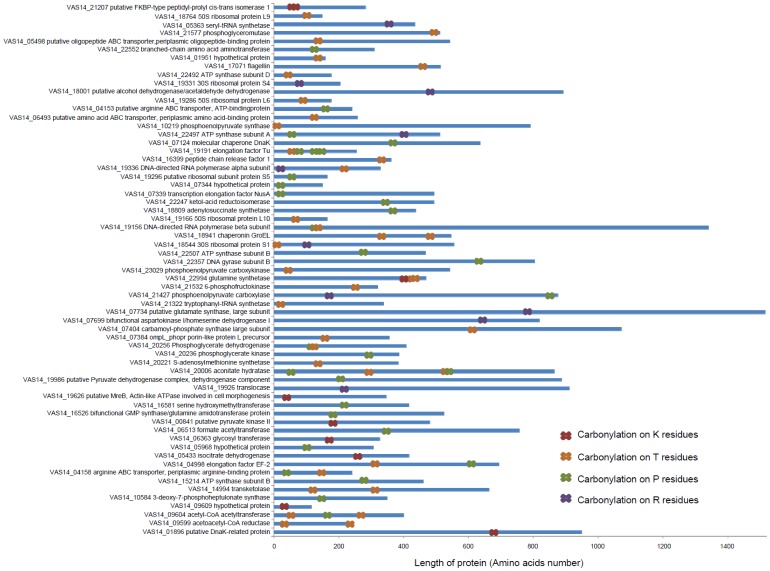
Location of carbonylated residues within the 62 proteins identified by DNPH derivatization.

The location of carbonylated residues was determined (Figure 4) and proteins classified according to their length: small (<200 amino acids), medium (200 to 600 amino acids), and long proteins (>600 amino acids). While no trend was discernible for the location of carbonylation in small and medium proteins, the 16 long proteins were almost exclusively oxidized in their N-terminal region or in the middle of their amino acids sequences, with only two proteins carbonylated in their C-terminal region (Figure 4). Sixteen long and medium proteins had multiple sites of carbonylation, and six proteins had three or more carbonylated amino acids. Of these six, three proteins (VAS14_22994 glutamine synthetase, VAS14_19191 elongation factor Tu, VAS14_20006 aconitate hydratase) exhibited a series of consecutive carbonylated amino acids. These regions were relatively rich in residues capable of being carbonylated (R, K, T or P).

**Figure 5:**
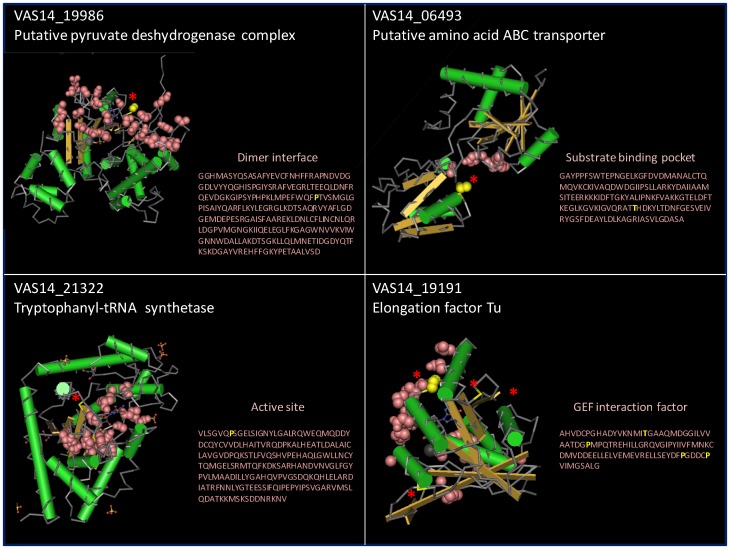
Predicted tertiary structures of proteins from *P. angustum* showing the location of carbonylation in functionally important domains. Pink balls represent the important functional domains. The location of carbonylation is shown in the protein structure (red stars) and primary amino acid sequence (yellow single letter code).

The location of the carbonyl groups was examined in the predicted tertiary structures of the proteins. Fifteen proteins were oxidized in functionally or structurally important domains such as substrate binding domains, active sites, and protein interaction interfaces (Tables 1, 2 bold proteins, Figure 5). These included carbonylation at T 141 located in the substrate binding pocket domain of a putative amino acid ABC transporter (VAS14_06493); P 191 in the dimer interface of a putative pyruvate dehydrogenase complex, dehydrogenase component (VAS14_19986); P 12 in the active site in tryptophanyl-tRNA synthetase (VAS14_21322); T 57 and P 75, 127, 132 in the guanine nucleotide exchange factor (GEF) interaction domain of Elongation Factor Tu (VAS14_19191) (Figure 5).

## Discussion

The goal of this study was to use redox proteomics to characterize proteins from the UVB resistant marine bacterium, *P. angustum,* that were oxidized *in vivo* during growth in the dark and those induced by UVB. In achieving this goal a new method using DNPH derivatization was developed, leading to the determination of what functional classes of proteins were carbonylated, which residues were preferentially modified, and based on the location of carbonylated sites within predicted tertiary structures, what the relevance of the carbonylation was likely to be for enzyme activity/protein function. The DNPH derivatization also proved to be useful for determining the proportion of cellular proteins that were carbonylated; an outcome that cannot be achieved using enrichment methods. Moreover, all the UVB biomarkers identified in a previous proteomic study that used 2D DIGE and ICPL [20], were identified in the present study, including RecA which had the highest level of up-regulation in both studies (ratio = 4.83, p value = 0.016 in this study and ratio = 3, Table 3 from reference 20), indicating that DNPH derivatization did not have any negative consequences for protein identification.

### Limitations of Biotin-avidin Enrichment

The biotinylation enrichment may have proven useful for determining which proteins were carbonylated. However, the approach was generally not useful for determining which amino acid residues were carbonylated as only three biotinylated peptides representing three proteins were identified. A previous study reported similar problems, failing to identify any carbonylated sites following enrichment using peptides or intact proteins [25]. Reports of the successful identification of biotinylated residues on carbonylated peptides are limited, and have only been described in studies in which large doses of external oxidizing agents have been used [35] or proteins were carbonylated *in vitro* and protein quantity was not limiting [36]. We speculate that problems with the approach may relate to steric hindrance of the biotin tag on trypsin cleavage site residues (arginine and lysine) resulting in the generation of long length peptides unsuitable for analysis in LC-MS/MS. Proteins enriched on the avidin column could also include endogenously biotinylated proteins such as a putative acetyl-CoA carboxylase (VAS14_19016), proteins with affinity for avidin, and/or proteins that co-purify (e.g. in complexes) with biotinylated proteins. Acetyl-CoA carboxylase is a biotin-dependent fatty acid biosynthesis enzyme that catalyzes the carboxylation of acetyl-CoA to produce malonyl-CoA, and has previously been purified on a sepharose-avidin column [37].

### Protein Structural Characteristics of Carbonylation

In *P. angustum* the rank order of amino acid carbonylation was P = T>R>K. The high relative abundance of proline carbonylation is consistent with a previous report [14]. However, the high relative abundance for threonine contrasts with speculation that threonine would be less susceptible because it is the least hydrophilic of amino acids capable of being carbonylated [38] and therefore was expected to be relatively buried in the protein [14]. Our data indicate that, at least for *P. angustum* proteins, this rationale is not correct. The three proteins VAS14_22994 glutamine synthetase, VAS14_19191 elongation factor Tu, and VAS14_20006 aconitate hydratase, contained consecutive carbonylated residues, but no recognizable motifs or compositional characteristics appeared to denote sites of carbonylation. The co-occurence of the amino acids RKTP (of which P is essential and at least two of three must be R, K or T) which has been reported to be a hotspot for carbonylation [11] was not diagnostic for predicting the sites that were carbonylated in *P. angustum*.

A total of 15 *P. angustum* proteins possessed at least one carbonylated residue located in a predicted key functional domain (Figure 5, Tables 2 and 3). Carbonylated proline was particularly abundant in these regions. As proline carbonylation often leads to cleavage of the polypeptide sequence, it is very likely to destroy protein function. Other studies have reported carbonylation preferentially occurring on protein surfaces due to favourable hydropathy [35], and protein surfaces may also be expected to be more often exposed to ROS via contact with oxidatively damaged molecules such as lipids, or oxidized metals. While it is clear that carbonylation generally leads to loss of enzymatic function [39], our study shows that a cause of loss of function can include the carbonylation of residues in important functional domains, some of which (*e.g.* VAS14_19986 pyruvate dehydrogenase, VAS14_06493_putative amino acid ABC transporter, VAS14_19191 elongation factor Tu) are on the surface of the protein (Figure 5).

### Biological Processes Associated with Carbonylated Proteins

Protein targets affected by carbonylation when *P. angustum* cells were grown under UVB radiation were alcohol dehydrogenase E, elongation factors (EF, Tu, NusA), chaperonins (GroEL, DnaK), ATPase, DNA gyrase, DNA directed RNA polymerases, and outer membrane protein (OmpL) (Table 2). The same types of proteins were also found to be carbonylated in *E. coli* when cells were submitted to different stress conditions such as hydrogen peroxide, superoxide-generating compounds, and iron excess [4]. In addition, they were detected in *E. coli* at the onset of stationary phase during carbon- or nitrogen-limited growth where they represented half of the total number of carbonylated proteins identified [39]. UVB induced carbonylation of proteins involved in metabolism, transcription, transport/folding and protein synthesis may therefore be the cellular functions that are most often affected by stress induced carbonylation, at least in certain bacteria.

**Figure 6:**
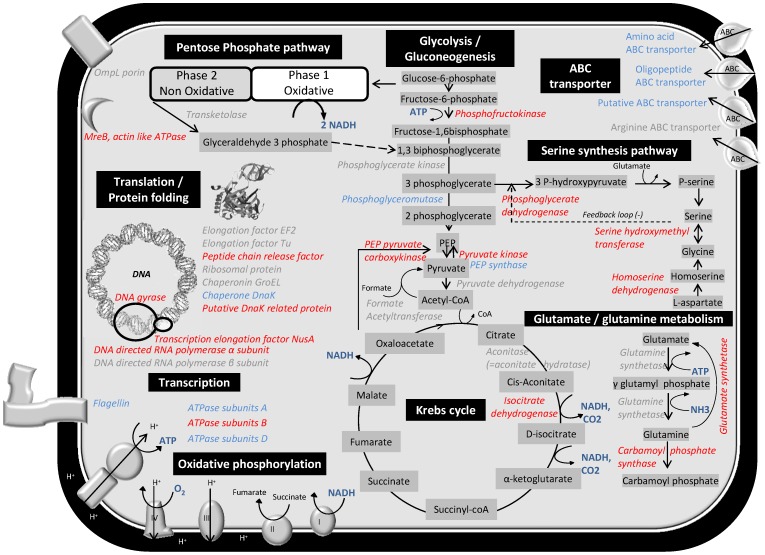
Diagram depicting the cellular pathways found to be carbonylated in *P. angustum*. Proteins carbonylated by UVB treatment (red), proteins carbonylated during dark treatment (blue), proteins carbonylated by both UVB and dark treatments (grey).

A total of nine proteins associated with Glycolysis or the Krebs Cycle were carbonylated during UVB or dark growth: phosphofructokinase (VAS14_21532), phosphoglycerate kinase (VAS14_20236), phosphoglyceromutase (VAS14_21577), pyruvate kinase (VAS14_00841), pyruvate dehydrogenase (VAS14_19986), phosphoenolpyruvate carboxylase (VAS14_21427), aconitate hydratase/aconitase (VAS14_20006), phosphoenol pyruvate carboxykinase (VAS14_23029), phosphoenolpyruvate synthase (VAS14_10219) (Tables 1 and 2, Figure 6). Aconitase, which functions in the Krebs Cycle to isomerize citrate to isocitrate, contains an Fe-S center in its active site and has been found to be particularly sensitive to oxidation, with superoxide causing the release of one iron atom [40], [41]. As a result, aconitase could indirectly cause carbonylation through metal-dependent oxidations [39], and enzymes involved in the Krebs Cycle in mitochondria of plant cells have previously been reported to be carbonylated [42]. Reduced activation of the Krebs Cycle leads to an increase in NAD accumulating and might alter the electron flow to oxygen, altering the redox homeostasis in the cell and thus indirectly enhancing oxidative stress. Collectively these data indicate that enzymes involved in some pathways leading to energy generation and reducing potential are generally sensitive to carbonylation.

The detection of carbonylation in three intracellular enzymes involved in serine synthesis and four in the glutamine/glutamate pathway only following UVB treatment, versus carbonylation in only one of these types of enzymes during dark growth (Tables 1 and 2, Figure 6) may indicate that these pathways are also susceptible to carbonylation by UVB. However, it is possible that the pathways are less critical to cell function during UVB stress and carbonylated proteins are not actively replaced.

Three DNA-binding proteins (DNA gyrase and two DNA polymerases; Table S3, Figure 6) were carbonylated only during UVB treatment, but none of the proteins function in DNA-repair, such as RecA. These findings are consistent with UVB treatment (at the dosage we employed) causing the upregulation of repair mechanisms that ensure that critical processes remain functional; in this case, mechanisms for repairing UVB induced DNA damage [20].

Three chaperones (VAS14_18941 chaperonin GroEL, VAS14_07124 molecular chaperone DnaK, VAS14_01896 putative DnaK related protein) were carbonylated. GroEL can function to refold unfolded proteins, as well as fold newly synthesized proteins. Interaction with unfolded proteins that have become oxidized may lead to GroEL being carbonylated, and GroEL itself may also become unfolded and subsequently carbonylated. These speculations are consistent with GroEL tending to be an abundant cellular protein in bacteria, and isomeric and cleaved forms being detected in numerous bacteria including a marine bacterium [43]. In addition to these chaperones, a number of proteins involved in transcription and translation were carbonylated, with similar numbers of proteins from UVB (13) and dark (10) treatments (Tables 2 and 3). A previous study reported that these types of proteins were up-regulated by UVB stress [20]. Taken together these findings may indicate that mistranslated or misfolded proteins, including defense proteins themselves (*e.g.* GroEL), become more susceptible to oxidation as aberrant oxidized proteins accumulate. This could function as a feedback mechanism [44], which in *P. angustum* operates effectively to maintain the total level of carbonylation at a level that does not compromise overall cell function (also see *Towards a model of carbonylation in P. angustum* below).

### Towards a Model of Carbonylation in *P. angustum*

The ability of *P. angustum* to control the extent of carbonylation is evidenced by the proportion of proteins that were carbonylated (5%) relative to the proportion of residues that are capable of being carbonylated (20% R+K+T+P). In fact, in the 62 carbonylated proteins only 92 residues were carbonylated (1.45% of 6337 R+K+T+P residues) indicating that the locations of carbonylation were very specific. Redox proteomics studies as comprehensive as ours are not available for other bacteria so we were not able to evaluate whether the extent of protein carbonylation as a proportion of total protein-encoding gene content in *P. angustum*, is typical. Therefore while our study serves as benchmark for future studies, we cannot determine if 5% is low and therefore indicative of an efficient protection mechanism. However, the fact that the proportion of carbonylation does not greatly increase when cells are exposed to UVB (45 for UVB and 36 for dark growth; Tables 2 and 3) does indicate that the cell is able to prevent a large increase in carbonylation from accumulating as a result of UVB damage. It is possible that UVB treatment upregulates the turnover of oxidatively damaged proteins including those that are carbonylated, aggregated or cleaved, whereas under less stressful conditions (in the dark) a background level of carbonylation is more easily tolerated.

For the 11 carbonylated proteins that were fully quantitated (Table 4), only one (OmpL_phopr porin-like protein L precursor) showed increased abundance and increased carbonylation following UVB treatment. OmpL is an outer membrane protein and it is possible that as a membrane protein it is more susceptible to damage and more difficult to replace in the membrane. As membrane proteins represented a somewhat larger proportion of proteins that were found to be carbonylated only during dark treatment (Table S3), this may indicate that during dark growth the consequences of oxidative damage to membrane proteins are able to be tolerated due to the overall low stress levels in the cell. Observing a reduced number of carbonylated membrane proteins during UVB also implies that defense mechanisms have been upregulated. In view of these results, in our model, carbonylation that occurs to key enzymes/proteins and/or occurs above a tolerable threshold leads to upregulation of defense systems. In support of this type of response, carbonylation functioning as a trigger to induce protein turnover and feedback control mechanisms have been reported for *E. coli* [10], [44], [45], [46], [47], and may function in a similar way to ubiquitinylation/proteasome systems in eukaryotes [10].

Our model also accommodates the likelihood that protein carbonylation is not only a consequence of oxidative stress, but functions as a signal (*e.g.* carbonylated proteins being more susceptible to proteolysis) to increase protein turnover (*e.g.* tagged for proteolytic degradation) and maintain an effective level of functional proteins. This would seem to be the case for glutamine synthetase, which was detected as a carbonylated protein with multiple sites of carbonylation from both UVB and dark treatments (Figure 4, Table S3). Studies on glutamine synthetase were some of the first to identify a link between oxidative modification that caused loss of catalytic activity, to lead to proteolytic degradation of the enzyme [45], with carbonyl groups specifically triggering degradative pathways [46], [47]. Regulation of the extent and nature of carbonylation in *P. angustum* therefore appears to involve pathways that control the extent of carbonylation acting as a signal for protein turnover, and mechanisms that function to defend against and respond to the presence of carbonylation.

### Conclusion

The DNPH derivatization approach involving LC-MS/MS proved powerful for determining the identity and quantity of carbonylated proteins, and the location of the modified residues, and is the first large scale, shotgun redox proteomics analysis performed on bacteria. The development of DNPH redox proteomics should now facilitate studies of carbonylation in other biological systems. Having established that an ecologically relevant dose of UVB does not lead to an increase in the extent of carbonylation in the cell, we can now use artificially higher doses of UVB to determine how the redox proteome changes, and establish how the changes relate to mechanisms of protection and cell survivability.

## Supporting Information

Table S1 **Carbonylated proteins labeled with biotin identified from UVB treated cells (141 proteins).**
(PDF)

Table S2 **Carbonyl proteins labeled with biotin identified from cells exposed to dark treatment (58 proteins).**
(PDF)

Table S3 **Carbonylated proteins identified after labeling with DNPH (62 non-redundant proteins).**
(PDF)
