# Supplementary material for: Shotgun Redox Proteomics: Identification and Quantitation of Carbonylated Proteins in the UVB-Resistant Marine Bacterium, Photobacterium angustum S14
Source: PLoS One. 2013 Jul 9;8(7):e68112. doi: 10.1371/journal.pone.0068112 (PMC3706606; doi:10.1371/journal.pone.0068112)
Supplement: Table S1 — Carbonylated proteins labeled with biotin identified from UVB treated cells (141 proteins). (PDF) [file pone.0068112.s001.pdf]

Table S1. Carbonylated proteins labeled with biotin identified from UVB treated cells (141 proteins).

Proteins name	COG	Protein score	*Nb of peptides	Sequence of carbonylated peptides
VAS14_07124 molecular chaperone DnaK	O	2662	64	
VAS14_20961 elongation factor Ts	J	2111	43	
VAS14_18544 30S ribosomal protein S1	J	2012	57	
VAS14_06218 trigger factor	O	1389	27	
VAS14_09609 hypothetical protein	/	1273	29	
VAS14_02983 putative Cold shock-like protein	K	1089	13	
VAS14_19171 50S ribosomal protein L1	J	1054	21	
VAS14_20441 phosphopyruvate hydratase	G	1025	24	
VAS14_19336 DNA-directed RNA polymerase alpha subunit	K	949	27	
VAS14_19161 50S ribosomal protein L7/L12	J	912	27	
VAS14_18941 chaperonin GroEL	O	835	22	
VAS14_07384 ompL_phopr porin-like protein L precursor	M	724	14	
VAS14_16921 putative cysteine synthase A	E	638	13	
VAS14_19321 30S ribosomal protein S13	J	617	15	
VAS14_05968 hypothetical protein	/	603	13	
VAS14_07334 translation initiation factor IF-2	J	555	17	
VAS14_09604 acetyl-CoA acetyltransferase	I	550	15	
VAS14_12874 hypothetical outer membrane protein OmpA	M	475	15	
VAS14_17071 flagellin	N	471	7	
VAS14_21577 phosphoglyceromutase	G	460	13	
VAS14_19331 30S ribosomal protein S4	J	434	10	
VAS14_18614 putative peptidyl-prolyl cis-trans isomerase	O	368	5	
VAS14_07339 transcription elongation factor NusA	K	349	8	
VAS14_19191 elongation factor Tu	J	349	7	
VAS14_19306 50S ribosomal protein L15	J	346	8	
VAS14_19266 50S ribosomal protein L24	J	346	9	
VAS14_20601 50S ribosomal protein L19	J	342	9	
VAS14_19206 50S ribosomal protein L3	J	335	7	
VAS14_21011 (3R)-hydroxymyristoyl ACP dehydratase	I	318	7	
VAS14_17676 ferric uptake regulator	P	310	6	
VAS14_18779 30S ribosomal protein S6	J	280	5	
VAS14_01741 integration host factor alpha subunit	L	270	7	
VAS14_21177 30S ribosomal protein S7	J	260	4	
VAS14_19341 50S ribosomal protein L17	J	246	7	
VAS14_06688 ferredoxin	C	239	5	
VAS14_19296 putative ribosomal subunit protein S5	J	238	5	
VAS14_09599 acetoacetyl-CoA reductase	IQR	231	6	
VAS14_21537 Hypothetical periplasmic protein CpxP	UNTP	226	3	
VAS14_19231 30S ribosomal protein S19	J	219	5	
VAS14_19511 single-strand DNA-binding protein	L	214	4	
VAS14_10584 3-deoxy-7-phosphoheptulonate synthase	E	200	4	
VAS14_19286 50S ribosomal protein L6	J	196	4	
VAS14_17896 putative DNA-binding protein H-NS	R	195	7	
VAS14_07379 transcription elongation factor GreA	K	193	7	
VAS14_22954 hypothetical protein	S	193	6	
VAS14_08295 50S ribosomal protein L21	J	188	6	
VAS14_11709 putative lipoprotein	/	184	4	
VAS14_03683 50S ribosomal protein L25	J	181	4	
VAS14_05238 acyl carrier protein	IQ	175	4	
VAS14_04158 arginine ABC transporter, periplasmic arginine-binding protein	ET	171	2	
VAS14_04998 elongation factor EF-2	J	161	4	
VAS14_19911 cell division protein FtsZ	D	161	4	
VAS14_19276 30S ribosomal protein S14	J	159	2	
VAS14_03053 putative antioxidant	O	153	4	
VAS14_18494 putative DNA topoisomerase (ATP-hydrolyzing)	L	151	5	
VAS14_10389 hypothetical protein	S	148	4	
VAS14_20761 putative DNA damage-inducible protein in SOS regulon	S	145	3	
VAS14_19201 30S ribosomal protein S10	J	143	3	
VAS14_01951 hypothetical protein	S	133	1	
VAS14_19176 50S ribosomal protein L11	J	133	2	
VAS14_13794 hypothetical protein	/	129	4	
VAS14_19281 30S ribosomal protein S8	J	127	3	
VAS14_05243 3-ketoacyl-(acyl-carrier-protein) reductase	IQR	126	3	
VAS14_18946 co-chaperonin GroES	O	124	3	
VAS14_15639 hypothetical protein	/	122	2	
VAS14_06783 putative antioxidant, AhpC/Tsa family protein	O	117	4	
VAS14_19236 50S ribosomal protein L22	J	116	6	
VAS14_20586 putative ribosomal protein S16	J	109	5	
VAS14_16916 phosphocarrier protein HPr	G	107	2	
VAS14_20496 recombinase A	L	103	3	
VAS14_16906 glucose-specific PTS system enzyme IIA component	G	101	2	

VAS14_08695 thiol peroxidase	O	101	2	
VAS14_18834 RNA-binding protein Hfq	R	99	3	
VAS14_19291 50S ribosomal protein L18	J	98	3	
VAS14_21001 putative outer membrane protein OmpH	M	97	3	
VAS14_19786 50S ribosomal protein L13	J	96	3	
VAS14_19241 30S ribosomal protein S3	J	95	1	
VAS14_19016 putative acetyl-CoA carboxylase, biotin carboxyl carrier protein	I	94	1	
VAS14_16911 Putative phosphoenolpyruvate-proteinphosphotransferase	U	91	1	
VAS14_21567 export protein SecB	U	91	3	
VAS14_18769 30S ribosomal protein S18	J	90	2	
VAS14_20951 methionine aminopeptidase	J	89	2	
VAS14_08105 30S ribosomal protein S21	J	89	3	
VAS14_06013 glyceraldehyde-3-phosphate dehydrogenase	G	84	2	
VAS14_08020 outer membrane channel precursor protein	MU	81	2	
VAS14_00581 leucyl-tRNA synthetase	J	81	1	
VAS14_22487 ATP synthase subunit B	C	78	1	
VAS14_19211 50S ribosomal protein L4	J	78	2	
VAS14_08120 RNA polymerase sigma factor	K	77	2	
VAS14_07629 hypothetical protein	O	75	2	
VAS14_00681 bacterioferritin comigratory protein	O	74	1	
VAS14_14989 transaldolase	G	74	2	
VAS14_18916 elongation factor P	J	72	2	
VAS14_08310 malate dehydrogenase	C	71	1	
VAS14_06208 ATP-dependent protease ATP-binding subunit	O	70	2	
VAS14_16581 serine hydroxymethyltransferase	E	69	2	VLDICK
VAS14_21222 peptidyl-prolyl cis-trans isomerase	O	68	2	
VAS14_14319 hypothetical protein		68	1	
VAS14_20516 carbon storage regulator	T	66	1	
VAS14_07970 Putative translation initiation inhibitor	J	65	1	
VAS14_18764 50S ribosomal protein L9	J	65	1	
VAS14_21076 putative lipoprotein YaeC	P	64	1	
VAS14_04723 putative heat shock protein HslJ	O	64	1	
VAS14_01246 hypothetical protein	P	64	2	
VAS14_17761 succinyl-CoA synthetase subunit beta	C	59	2	
VAS14_21182 30S ribosomal protein S12	J	58	1	
VAS14_08340 inorganic pyrophosphatase	C	57	1	
VAS14_20201 glutathione synthetase	HJ	56	1	
VAS14_05963 hypothetical outer membrane protein OmpA	M	55	1	
VAS14_16399 peptide chain release factor 1	J	54	1	
VAS14_19221 50S ribosomal protein L2	J	54	1	
VAS14_22392 50S ribosomal protein L34	J	53	2	
VAS14_22197 peptide ABC transporter, periplasmic peptide-binding protein	E	52	1	
VAS14_06198 putative bacterial nucleoid DNA-binding protein	L	51	1	
VAS14_17571 hypothetical protein	S	51	1	
VAS14_02181 hypothetical protein	/	50	1	
VAS14_07684 putative formate acetyl transferase-related protein	R	48	1	
VAS14_17541 OmpH porin-like protein H precursor	M	47	2	
VAS14_21362 shikimate kinase I	E	47	1	
VAS14_08290 50S ribosomal protein L27	J	46	1	
VAS14_20236 phosphoglycerate kinase	G	45	1	
VAS14_21667 putative ribosomal protein L28	J	45	1	
VAS14_06708 putative NifU-related protein	C	43	1	
VAS14_21277 putative cyclic AMP receptor protein	T	43	1	
VAS14_00453 uracil phosphoribosyltransferase	F	40	1	
VAS14_07945 hypothetical protein	S	40	1	
VAS14_18824 putative Membrane protease subunits	O	39	1	
VAS14_22552 branched-chain amino acid aminotransferase	EH	37	1	
VAS14_21457 50S ribosomal protein L31	J	36	1	
VAS14_19256 30S ribosomal protein S17	J	35	1	
VAS14_11494 hypothetical protein	/	34	1	
VAS14_22507 ATP synthase subunit B	C	34	1	
VAS14_14879 hypothetical protein	S	34	1	
VAS14_18744 hypothetical protein	M	34	1	
VAS14_20556 glutamate--cysteine ligase	H	33	1	
VAS14_03193 hypothetical protein	/	33	1	
VAS14_07449 putative ribosomal protein S20	J	33	1	
VAS14_07314 polyribonucleotide nucleotidyltransferase	J	33	1	
VAS14_17596 adenylate kinase	S	33	1	
VAS14_04308 hypothetical protein	S	33	1	
VAS14_07649 hypothetical O-methyltransferase	R	32	1	LLLQLSK

* Nb of peptides: number of non-redundant peptides, identification using Mascot. (Mass spectrometry: short run)
