# Supplementary material for: Shotgun Redox Proteomics: Identification and Quantitation of Carbonylated Proteins in the UVB-Resistant Marine Bacterium, Photobacterium angustum S14
Source: PLoS One. 2013 Jul 9;8(7):e68112. doi: 10.1371/journal.pone.0068112 (PMC3706606; doi:10.1371/journal.pone.0068112)
Supplement: Table S2 — Carbonyl proteins labeled with biotin identified from cells exposed to dark treatment (58 proteins). (PDF) [file pone.0068112.s002.pdf]

Table S2. Carbonyl proteins labeled with biotin identified from cells exposed to dark treatment (58 proteins).

Protein name	COG	Protein score	*Nb of peptides	Sequence of carbonylated peptides
VAS14_20961 elongation factor Ts	J	2333	42	
VAS14_06218 trigger factor	O	2209	44	
VAS14_07124 molecular chaperone DnaK	O	1845	37	
VAS14_12874 hypothetical outer membrane protein OmpA	M	1324	34	
VAS14_09359 cold shock protein	K	1071	13	
VAS14_19171 50S ribosomal protein L1	J	1004	24	
VAS14_18544 30S ribosomal protein S1	J	941	22	
VAS14_17071 flagellin	N	937	16	
VAS14_07384 ompL_phopr porin-like protein L precursor	M	699	14	
VAS14_05968 hypothetical protein	/	555	11	
VAS14_16916 phosphocarrier protein HPr	G	499	13	
VAS14_18614 putative peptidyl-prolyl cis-trans isomerase	O	484	8	
VAS14_19161 50S ribosomal protein L7/L12	J	461	12	
VAS14_04158 arginine ABC transporter	ET	443	9	
VAS14_19321 30S ribosomal protein S13	J	417	8	
VAS14_09609 hypothetical protein	/	412	10	
VAS14_18779 30S ribosomal protein S6	J	407	5	
VAS14_19266 50S ribosomal protein L24	J	394	10	
VAS14_08290 50S ribosomal protein L27	J	247	9	
VAS14_20601 50S ribosomal protein L19	J	231	4	
VAS14_11709 putative lipoprotein	/	211	4	
VAS14_19336 DNA-directed RNA polymerase alpha subunit	K	173	4	
VAS14_19281 30S ribosomal protein S8	J	153	6	
VAS14_19206 50S ribosomal protein L3	J	152	3	
VAS14_16906 glucose-specific PTS system enzyme IIA component	G	132	2	
VAS14_18946 co-chaperonin GroES	O	128	3	
VAS14_18764 50S ribosomal protein L9	J	119	2	
VAS14_05238 acyl carrier protein	IQ	115	2	
VAS14_04308 hypothetical protein	S	98	2	
VAS14_07329 ribosome-binding factor A	J	89	1	
VAS14_08295 50S ribosomal protein L21	J	88	1	
VAS14_04258 putative amino acid ABC transporter	ET	87	1	
VAS14_01951 hypothetical protein	S	77	1	
VAS14_22197 peptide ABC transporter	E	77	1	
VAS14_17896 putative DNA-binding protein H-NS	R	74	2	
VAS14_19286 50S ribosomal protein L6	J	72	3	
VAS14_18051 hypothetical protein	S	70	1	
VAS14_07970 Putative translation initiation inhibitor	J	63	1	
VAS14_19511 single-strand DNA-binding protein	L	61	1	
VAS14_20971 ribosome releasing factor	J	60	1	
VAS14_16921 putative cysteine synthase A	E	58	1	
VAS14_19191 elongation factor Tu	J	54	1	
VAS14_14319 hypothetical protein	/	53	1	
VAS14_19306 50S ribosomal protein L15	J	51	1	
VAS14_06198 putative bacterial nucleoid DNA-binding protein	L	49	1	
VAS14_05653 putative superoxide dismutase	P	48	1	

VAS14_07945 hypothetical protein	S	45	1	
VAS14_08455 diadenosine tetraphosphatase	R	40	1	
VAS14_08695 thiol peroxidase	O	39	1	
VAS14_11299 putative glycine betaine-binding ABC transporter	E	38	1	
VAS14_19331 30S ribosomal protein S4	J	38	1	
VAS14_19341 50S ribosomal protein L17	J	37	1	
VAS14_21577 phosphoglyceromutase	G	35	1	
VAS14_17541 OmpH porin-like protein H precursor	M	34	1	
VAS14_03193 hypothetical protein	/	34	1	
VAS14_06493 putative amino acid ABC transporter	ET	34	2	
VAS14_19166 50S ribosomal protein L10	J	32	1	
VAS14_07649 hypothetical O-methyltransferase	R	30	1	LLLQLS**K**

[*] Nb of peptides: number of non-redundant peptides, identification using Mascot. (Mass spectrometry:short run)
