# Supplementary material for: Shotgun Redox Proteomics: Identification and Quantitation of Carbonylated Proteins in the UVB-Resistant Marine Bacterium, Photobacterium angustum S14
Source: PLoS One. 2013 Jul 9;8(7):e68112. doi: 10.1371/journal.pone.0068112 (PMC3706606; doi:10.1371/journal.pone.0068112)
Supplement: Table S3 — Carbonylated proteins identified after labeling with DNPH (62 non-redundant proteins). (PDF) [file pone.0068112.s003.pdf]

Table S3. Carbonylated proteins identified after labeling with DNPH (62 non-redundant proteins).

Carbonylated proteins only identified in UVB condition*	
VAS14_00841	putative pyruvate kinase II
VAS14_01896	putative DnaK-related protein
VAS14_05433	isocitrate dehydrogenase
VAS14_06363	glycosyl transferase
VAS14_07339	transcription elongation factor NusA
VAS14_07344	hypothetical protein
VAS14_07404	carbamoyl-phosphate synthase large subunit
VAS14_07699	bifunctional aspartokinase I / homeserine dehydrogenase I
VAS14_07734	putative glutamate synthase, large subunit
VAS14_09609	hypothetical protein
VAS14_15214	ATP synthase subunit B
VAS14_16399	peptide chain release factor 1
VAS14_16526	bifunctional GMP synthase/glutamine amidotransferase
VAS14_16581	serine hydroxymethyltransferase
VAS14_18544	30S ribosomal protein S1
VAS14_19166	50S ribosomal protein L10
VAS14_19336	DNA-directed RNA polymerase alpha subunit
VAS14_19626	putative MreB, Actin-like ATPase
VAS14_19926	translocase
VAS14_20221	S-adenosylmethionine synthetase
VAS14_20256	Phosphoglycerate dehydrogenase
VAS14_21322	tryptophanyl-tRNA synthetase
VAS14_21427	phosphoenolpyruvate carboxylase
VAS14_21532	6-phosphofructokinase
VAS14_22357	DNA gyrase subunit B
VAS14_23029	phosphoenolpyruvate carboxykinase
Carbonylated proteins only identified in Dark condition*	
VAS14_01951	hypothetical protein
VAS14_04153	putative arginine ABC transporter, ATP-binding protein
VAS14_05363	seryl-tRNA synthetase
VAS14_05498	putative oligopeptide ABC transporter
VAS14_06493	putative amino acid ABC transporter
VAS14_07124	molecular chaperone DnaK
VAS14_10219	phosphoenolpyruvate synthase
VAS14_17071	flagellin
VAS14_18001	putative alcohol dehydrogenase/acetaldehyde dehydrogenase
VAS14_18764	50S ribosomal protein L9
VAS14_19286	50S ribosomal protein L6
VAS14_19331	30S ribosomal protein S4
VAS14_21207	putative FKBP-type peptidyl-prolyl cis-trans isomerase 1
VAS14_21577	phosphoglyceromutase
VAS14_22492	ATP synthase subunit D
VAS14_22497	ATP synthase subunit A
VAS14_22552	branched-chain amino acid aminotransferase
Carbonylated proteins identified in both UVB and Dark conditions*	
VAS14_04158	arginine ABC transporter
VAS14_04998	elongation factor EF-2
VAS14_05968	hypothetical protein
VAS14_06513	formate acetyltransferase
VAS14_07384	ompL_phopr porin-like protein L precursor
VAS14_09599	acetoacetyl-CoA reductase
VAS14_09604	acetyl-CoA acetyltransferase
VAS14_10584	3-deoxy-7-phosphoheptulonate synthase
VAS14_14994	transketolase
VAS14_18809	adenylosuccinate synthetase
VAS14_18941	chaperonin GroEL
VAS14_19156	DNA-directed RNA polymerase beta subunit
VAS14_19191	elongation factor Tu
VAS14_19296	putative ribosomal subunit protein S5
VAS14_19986	putative Pyruvate dehydrogenase complex
VAS14_20006	aconitate hydratase
VAS14_20236	phosphoglycerate kinase
VAS14_22247	ketol-acid reductoisomerase
VAS14_22994	glutamine synthetase

* Protein identification using Protein Pilot / Mass spectrometry: long run.
